# Widespread genetic heterogeneity and genotypic grouping associated with fungicide resistance among barley spot form net blotch isolates in Australia

**DOI:** 10.1093/g3journal/jkad076

**Published:** 2023-04-01

**Authors:** Kealan Hassett, Mariano Jordi Muria-Gonzalez, Aleesha Turner, Mark S McLean, Hugh Wallwork, Anke Martin, Simon R Ellwood

**Affiliations:** Centre for Crop and Disease Management, Curtin University, Bentley, WA 6102, Australia; Centre for Crop and Disease Management, Curtin University, Bentley, WA 6102, Australia; Centre for Crop and Disease Management, Curtin University, Bentley, WA 6102, Australia; Field Crops Pathology, Agriculture Victoria, Horsham, Victoria 3401, Australia; Cereal Pathology Laboratory, South Australian Research and Development Institute, Hartley Grove, Urrbrae, SA 5064, Australia; Centre for Crop Health, University of Southern Queensland, Toowoomba, Queensland 4350, Australia; Centre for Crop and Disease Management, Curtin University, Bentley, WA 6102, Australia

**Keywords:** *Hordeum vulgare*, fungal plant pathogen, diversity arrays technology, necrotroph

## Abstract

Spot form net blotch, caused by *Pyrenophora teres* f. *maculata*, is a major foliar disease of barley worldwide. Knowledge of the pathogen's genetic diversity and population structure is critical for a better understanding of inherent evolutionary capacity and for the development of sustainable disease management strategies. Genome-wide, single nucleotide polymorphism data of 254 Australian isolates revealed genotypic diversity and an absence of population structure, either between states, or between fields and cultivars in different agro-ecological zones. This indicates there is little geographical isolation or cultivar directional selection and that the pathogen is highly mobile across the continent. However, two cryptic genotypic groups were found only in Western Australia, predominantly associated with genes involved in fungicide resistance. The findings in this study are discussed in the context of current cultivar resistance and the pathogen's adaptive potential.

## Introduction

Spot form net blotch (SFNB) is a major foliar disease of barley worldwide (Liu *et al*. 2011), caused by the ascomycete fungus *Pyrenophora teres* f. *maculata* (*Ptm*) (Smedegård-Petersen 1971). The pathogen is morphologically similar, yet phylogenetically distinct from *Pyrenophora teres* f. *teres* (*Ptt*), the causal agent of net form net blotch (NFNB) disease. The two diseases are distinguished by their physiological leaf symptoms, with SFNB characterized by round brown lesions surrounded by a yellow chlorotic halo and NFNB characterized by dark brown, net-like necrotic lesions striate along barley leaf veins. Despite occurring on the same host and their similar morphological characteristics, the two diseases are treated separately as they interact with different host resistance and susceptibility genes (Clare *et al*. 2020) and, although natural hybridization is possible, this is rare (Poudel *et al*. 2017). In Australia, SFNB is of economic importance, with yield losses of up to 20% and an increase of up to 18% in undersized grain (McLean *et al*. 2022).

Fungal pathogen evolution in plants is broadly governed by effectors, small rapidly evolving secreted proteins or molecules, which interact with the host in different ways dependent on pathogen lifestyle (Plissonneau *et al*. 2017). For example, biotrophs secrete effectors that suppress host defenses and subvert metabolism to allow proliferation (Dodds *et al*. 2009). Necrotrophs, by contrast, secrete effectors that promote cell death (Faris and Friesen 2020). Host recognition and effector diversification drive the interaction between fungal pathogens and plants, with dominant host resistance against biotrophs leading to the loss or alteration of the corresponding virulence (effector) gene. In necrotrophs such as *Ptm*, there is a predominantly opposite genetic relationship, known as the inverse gene-for-gene model, where susceptibility is dominant as genes encoding host target products are lost or altered (Peters Haugrud *et al*. 2019; Muria-Gonzalez *et al*. 2023).

The genetic structure and diversity of *Ptm* has been previously assessed within Australia using both simple sequence repeats (SSRs, Bogacki *et al*. 2010; McLean *et al*. 2010) and amplified length polymorphism (AFLP) markers (Serenius *et al*. 2007; Lehmensiek *et al*. 2010; McLean *et al*. 2014). These studies analyzed modest numbers of both isolates (31–60) and polymorphic markers (15–109) and found low genetic differentiation between different regions and fields and no correlation with geographic origin, although both Serenius *et al*. (2007) and McLean *et al*. (2010) found differences in the levels of genetic diversity between regions.

Globally, genetic diversity studies of *Ptm* have been performed in Europe (Rau *et al*. 2003; Bakonyi and Justesen 2007), North America (Akhavan *et al*. 2016), Algeria (Ahmed Lhadj *et al*. 2022), Iran (Vasighzadeh *et al*. 2021), the Republic of South Africa (Campbell *et al*. 2002), and Turkey (Oğuz *et al*. 2019). In common with previous Australian studies, these employed older genetic marker technologies including SSRs, inter-simple sequence repeats, and AFLPs and generally found significant levels of sexual recombination and low levels of clonality. The studies in both Sardinia and Canada found no significant genetic differentiation between populations. In Iran, however, using the same SSRs as those in the Canadian study, higher genetic variation was apparent together with significant evidence for regional population structure, while Lehmensiek *et al*. (2010) were able to distinguish isolates from South Africa and Australia.

Although microsatellites and AFLP DNA markers have been commonly used for comparing individuals with high levels of genetic diversity, they usually lack high marker numbers (in the case of microsatellites) and precise genomic context and even genomic distribution (in the case of AFLPs) available with genotype-by-sequencing (GBS) techniques, where large, single nucleotide polymorphism (SNP) datasets are produced. GBS techniques have been shown to provide clearer detection of finer scale genetic structuring compared to microsatellite data (Sunde *et al*. 2020). Furthermore, hundreds or thousands of SNP markers generated throughout the target genome produce higher resolution data on smaller sample sizes compared to SSRs (Jeffries *et al*. 2016). This allows better comparisons between both strongly and weakly diverged populations (Andrews *et al*. 2016), as well as better inferences on population structure (Bruneaux *et al*. 2013). One popular method of generating SNP markers is by Diversity Arrays Technology (DArT), a highly parallel genome-wide approach based on a restriction enzyme complexity reduction step that selects for non-repetitive (coding) DNA (Sansaloni *et al*. 2011). The DArT system produces reproducible silicoDArT (presence-absence markers) and DArTseq (SNP) markers from genomic DNA extracts. This technology has been previously implemented for genetic mapping and genotypic analyses of fungal species including *P. teres* (Syme *et al*. 2018; Poudel *et al*. 2019).

We hypothesized that the composition of the *Ptm* population across Australia is likely to be influenced by geographic isolation and the cultivars (cvs) being grown, and that low sample numbers together with few or potentially clustered genetic markers in previous studies may have excluded genomic regions contributing to population structure. To assess this, and to provide greater resolution of the genetic diversity and the distribution of *Ptm* genotypes in Australia, we analyzed isolates based on DArTseq SNP markers. These were sampled at two levels: on the one hand isolates collected from across the major barley-growing regions and, on the other hand, isolates collected from six fields at three sites in Western Australia. The isolates were collected between 2017 and 2020, with the majority of Western Australian isolates collected in 2020. Their genetic diversity was then assessed, and the relationships between isolates were compared by Bayesian, multivariate, and geographic distance-based approaches.

## Methods

### Field-scale *Ptm* isolate collections

**Fig. 1.**
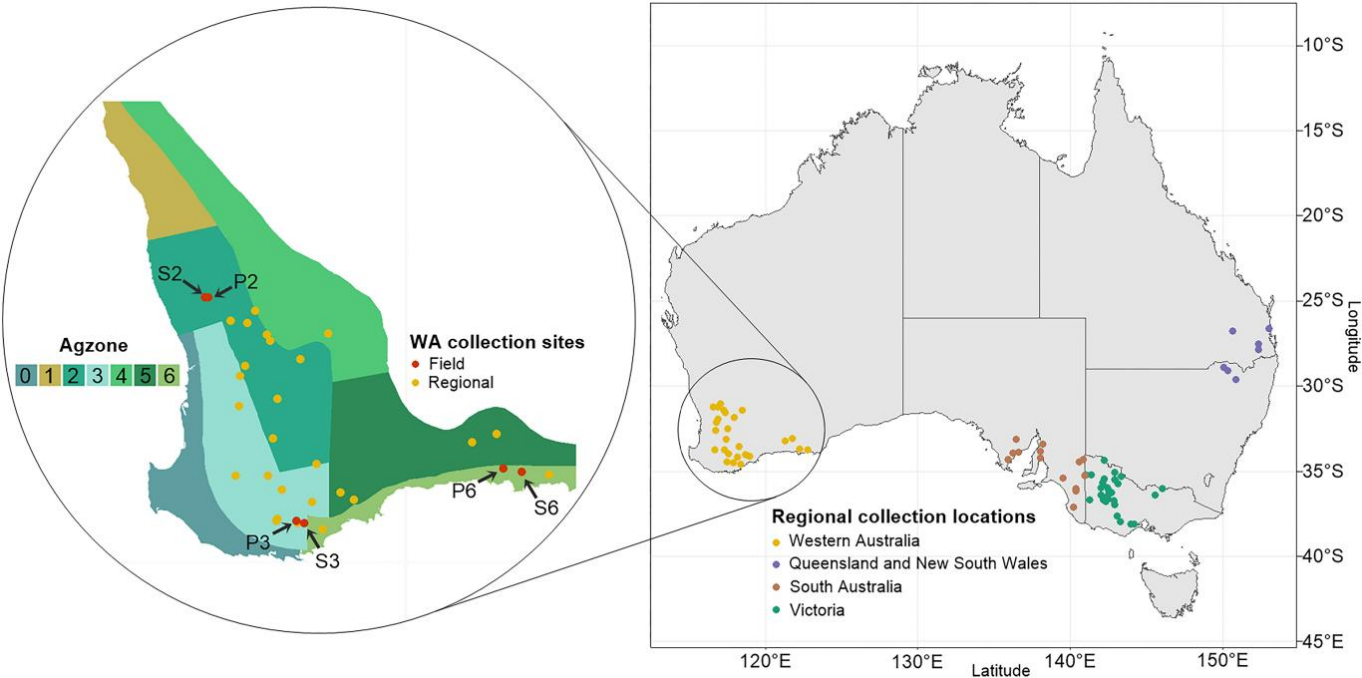
*Ptm* collection sites from Australian states and Western Australian Agzones. Six Western Australian fields from which diseased leaves were sampled are indicated with arrows. Agzone map data was obtained from Geographic Information Services, Perth, WA (2016).

Isolates were sampled from six fields across three agricultural zones (Agzones) of Western Australia (WA) for inter-field analyses (Supplementary Table 2, Fig. 1). Agzones 2, 3, and 6 were chosen due to their status as high rainfall zones (>450 mm per year) suited to barley growth, whilst spanning the majority of the barley-growing area of WA. Two fields per Agzone were sampled, one of barley cv Spartacus CL and the other of cv RGT Planet. In each field, samples were obtained from a 60 m × 100 m area divided into 100 m^2^ sections. Three leaves displaying SFNB symptoms were collected from each section and stored in paper envelopes.

### Inter-regional collections of *Ptm* isolates and related *Pyrenophora* sp.

To investigate national *Ptm* diversity, a total of 87 *Ptm* isolates were provided by the Centre of Crop and Disease Management, the South Australian Research and Development Institute and Agriculture Victoria (AgVic, Fig. 1). The isolates were from locations dispersed across the majority of major barley-growing regions of WA, South Australia (SA), Victoria (Vic), New South Wales (NSW), and Queensland (Qld). Included in the regional *Ptm* collections were four randomly chosen *Ptt* isolates from the same institutions.

### Fungal isolation

To obtain single-spored isolates, leaf samples were dried at room temperature for 2 weeks, then surface sterilized (30 seconds in 15% ethanol, followed by 30 seconds in 5% ethanol and 1% bleach, and rinsed two times for 30 seconds in sterile water). Net blotch-like lesions were excised and placed on a petri dish containing sterile paper towel wetted with sterile water, sealed with Parafilm (Bemis Inc., Neenah, WI, USA) and incubated at 18°C with a 14-hour photoperiod for up to 7 days. Leaves were inspected for conidia formation daily under a binocular microscope from the third day post plating. Cultures where no conidia were produced after 7 days were placed under near UV light for 18 hours at room temperature, followed by 24 hours in the dark at 15°C to stimulate sporulation. Mature conidia were collected with a sterile acupuncture needle and transferred to V8-PDA agar plates [150-ml/l V8 juice (Campbell's Soups Australia, Lemnos, VIC, Australia), 10-g/l agar (Oxoid Ltd., Basingstoke, UK), 10-g/l Difco potato dextrose agar (Becton Dickinson, Sparks, MD, USA), and 3-g/l CaCO3]. Plates were incubated at room temperature for 5 days before 4-mm^2^ plugs were cut from each colony, then air-dried in a biosafety cabinet overnight before storage at −80°C.

### DNA extraction and identification of *P. teres* mating type loci

Cultures were grown for 5 days in Fries 2 liquid medium (Fries 1938). The mycelia were lyophilized and genomic DNA isolated using a Promega Wizard Genomic DNA Purification Kit (Fitchburg, Wisconsin, USA) according to the “Isolating genomic DNA from plant tissue” protocol. DNA concentration and quality were measured with a NanoDrop spectrophotometer (Thermo Fisher Scientific, MA, USA). *MAT* (mating-type) locus mating type and *formae* were determined by the polymerase chain reaction (PCR) method according to Lu *et al*. (2010).

### Genotype-by-sequencing and DArTseq data filtering

*Pyrenophora teres* isolates were genotyped using DArTSeq whole genome sequencing at Diversity Arrays Technology Pty Ltd (Canberra, Australia). DArT is a highly parallel, genome-wide genotyping technology developed by Jaccoud *et al*. (2001) combined with next-generation sequencing (Sansaloni *et al*. 2011) and proprietary analytical pipelines (Ren *et al*. 2015). DArTSeq genome complexity reduction was performed with *Pst*I and *Mse*I restriction enzymes, followed by next-generation sequencing using a HiSeq2000 DNA sequencing platform (Illumina, USA). In total, 5110 DArTSeq (SNP markers) and 6321 silicoDArT (presence/absence markers) were produced. DArT genotype data is provided in Supplementary Table 1.

SNP markers were converted into the Genalex file format (Peakall and Smouse 2012) and imported into RStudio 4.2.0 (RStudio, Boston, MA, USA). Markers and isolates with >10% missing data were removed. Phylogenetically uninformative loci, those containing less than a given percentage of divergent individuals (cut off = 2/*n*) and minimum allele frequency (MAF = 0.01), were removed using the *informloci* command in the *poppr* package (Kamvar *et al*. 2014).

Unique multi-locus genotypes (MLGs) were determined by first establishing the genetic distance between two sets of replicate samples to compensate for SNP genotyping errors associated with DArTseq. The average genotyping error for DArTseq SNPs between pairs of template control DNA samples has been reported as averaging 0.8%, which is lower than other SNP genotyping platforms and DNA sequencing (Ndjiondjop *et al*. 2018). Genetic distances within both sets of biological replicates were calculated using the *provesti.dist* model, and used to filter the data with the *mlgfilter* command in the *poppr* package (default parameters), such that isolates sharing a genetic distance below the calculated value were considered the same MLG. Isolates within the same MLG were classified as clones using the *clonecorrect* function in *poppr*.

### Resolution of *P. teres* forms

Based on initial comparisons, DArTSeq SNP markers (rather than dominant silicoDArT markers) were chosen to assess the *formae* relationships, together with subsequent regional and field level analyses, due to the silicoDArT markers not adding novel information. SillicoDArT markers were analyzed separately, and generally supported the conclusions found with the DArT SNP markers (Supplementary File 1). An initial assessment of the effectiveness of DArT genotype data to distinguish *P. teres formae* isolates was conducted using a group of four *Ptt* isolates, including reference genome isolate W1–1, the *Ptm* reference isolate SG1 (Syme *et al*. 2018), and four randomly selected *Ptm* isolates (106/16A, 20P3001, PTM18-024, and 71/17, see Supplementary Table 2).

### Genetic diversity and linkage disequilibrium

The genetic diversity of groups of isolates was determined by comparing the number of MLGs to the expected number of MLGs in the original data, together with Simpson's corrected index ((*N*/(*N* − 1))*λ*) of multi-locus genotype diversity. To determine the extent of random mating occurring within populations, gametic equilibrium was calculated using the standardized index of association (*r̄*_d_), which is sample size independent (Agapow and Burt 2001), within the *poppr* package in Rstudio (RStudio, Boston, MA, USA).

### Analysis of molecular variance

In order to detect genetic variation within and between groups of isolates for each comparison (between forms, regions, and fields), an analysis of molecular variance (AMOVA) was carried out. Variance (*σ*) as well as the population differentiation statistics (*ϕ*) were calculated using the *poppr.amova* command in the *poppr* package.

### Principle components analysis and discriminant analysis of principle components

Principal component analysis (PCA), a multivariate statistical approach, was used to inform genetic clustering of individuals. In addition, genetic subdivision was inferred through discriminant analysis of principle components (DAPC), a method optimized for large datasets that like PCA does not rely on pre-existing genetic models but which can examine more complicated scenarios, detecting between-group variability and structures existing among clusters (Jombart *et al*. 2010). For the PCAs, clone-corrected data was investigated with the *glpca* function in the *adegenet* R package to observe the impact of eigenvalues on the overall variance explained (Jombart 2008). DAPC was performed after first observing the *K*-means clustering graph at the lowest Bayesian information criteria, using the *find.clusters* command in the *adegenet* package, and then applying the appropriate value for each of the DAPC analyses. DAPC was performed with the *dapc* command in *adegenet*, applying the result derived from the cross validation function *Xval.dapc* to confirm the correct number of PC to retain (Dray and Dufour 2007). The *snpzip* command using the “median” method in the *adegenet* package was then used to calculate the contribution (loadings) of each SNP to groups assigned in the clustering analysis. DArTSeq sequences (69 bp in length) responsible for genetic clusters were examined by BLAST at the National Center for Biotechnology Information and in Geneious 8.0.5 (Kearse *et al*. 2012) to find annotated homologous genes in *Ptm* or in related fungi including *Ptt* and *Pyrenophora tritici-repentis* (*Ptr*) (Supplementary Table 3).

### Bayesian inference-based clustering analysis

Genetic structure was also examined via a Bayesian inference-based method, implemented in STRUCTURE version 2.3.4 (Pritchard *et al*. 2000). STRUCTURE is a model-based clustering approach that assumes loci within populations are at Hardy–Weinberg equilibrium and linkage equilibrium, although allowing for admixture linkage disequilibrium within a population, enabling detection of subtle population subdivisions (Falush *et al*. 2003). SNP data was analyzed using a burn-in period of 100,000 steps and 100,000 replications, assessing *K* values between one and nine with 10 iterations. The STUCTURE output file data was then processed with STRUCTURE HARVESTER (Earl and vonHoldt 2011) to determine the optimal *K* value.

### Genetic isolation by distance

To detect the correlation between genetic distance and geographic distance, a Mantel test was carried out based on each isolate's sampling GPS coordinates or, in the absence of these, the closest known location. The *provesti.dist* function was used to produce a matrix of the genetic distances between individuals and used alongside the *dist* function in R to produce a distance matrix of the GPS coordinates. The *mantel.rtest* was used to calculate the likelihood of isolation by distance in the *ade4* package.

## Results

### Inter-*formae* SNP diversity and differentiation

In order to assess the accuracy of the DArTseq SNP dataset to differentiate the *P. teres* forms and their relative genetic relatedness, subsamples of *Ptm* and *Ptt* individuals were compared. After data filtering, 1594 SNP markers remained. Total SNP variance within *forma* was relatively low (7.7%), comprising only a small portion of the total observed variation, whereas variation between *formae* was high (92.3%) and comprised the majority of the total variation. This was complimented by AMOVA differentiation statistics between the two groups, which was high (*ϕ* = 0.92), while a PCA showed the first two principle components explained ∼90% of the variation.

### Field-level population structure of *Ptm* isolates

After filtering by allele call rate and missing data, 164 out of the initial 166 WA *Ptm* isolates remained, providing 11 to 36 isolates per field. Removing non-informative loci left 1252 SNP markers for further analysis. Total SNP variance within fields was high and comprised most of the total variation (≥99%), whereas variation between fields comprised ≤1% of the total. The isolates showed a high level of individual variation, grouping into 155 MLGs (Table 1), while population differentiation was low (*ϕ* = 0.0035). Nei's unbiased gene diversity, which indicates the probability that two randomly chosen alleles are different, was moderate for all populations (>0.196); and the corrected Simpson's index, which represents the probability of two random isolates drawn from a population to be of a different genotype, suggested high genotype diversity for all populations (1 − *λd* > 0.99). Despite the high genotype diversity, all fields appeared to be primarily reproducing asexually as suggested by the standardized index of association (*r̄*_d_) values (between 0.003 and 0.019, *P* = 0.01).

**Table 1.** Genetic diversity indices of Australian *Ptm* isolates.

	*n*	MLG	eMLG	*H*	1 − *λ*	*H*_exp_	r̄_d_
**ȃ**Field*^a^*							
ȃP2	27	27	11.0	3.30	1.00	0.21	1.5 × 10^−2^
ȃP3	36	36	11.0	3.58	1.00	0.20	3.0 × 10^−3^
ȃP6	11	11	11.0	2.40	1.00	0.20	5.0 × 10^−3^
ȃS2	28	26	10.7	3.23	0.99	0.20	1.1 × 10^−2^
ȃS3	27	27	11.0	3.30	1.00	0.20	3.0 × 10^−3^
ȃS6	35	32	10.7	3.42	0.99	0.20	1.9 × 10^−2^
ȃTotal	164	155*^b^*	10.9	5.00	1.00	0.21	8.8 × 10^−3^
Region							
ȃVic	30	30	10.0	3.40	1.00	0.20	9.2 × 10^−4^
ȃSA	20	20	10.0	3.00	1.00	0.21	1.5 × 10^−3^
ȃQld and NSW	8	8	8.0	2.08	1.00	0.19	3.4 × 10^−3^
ȃWA	29	27	9.7	3.25	0.99	0.21	6.7 × 10^−3^
ȃTotal	87	85	10.0	4.43	1.00	0.21	2.5 × 10^−3^

Data is presented for groups sampled within WA fields and across four regional areas, produced within the *poppr* package (Kamvar *et al*. 2014).

*n*: number of isolates in a sample group after data-quality filtering.

MLG: the total number of unique multi-locus genotypes (MLGs) identified per field or region.

eMLG: expected number of MLGs.

*H*: Shannon–Wiener index of MLG group genotypic diversity, a measure of the number of unique genotypes and their homogeneity.

1 − *λ*: corrected Simpson's index of MLG diversity, the probability that two isolates from the same dataset are different genotypes.

*H*_exp_: Nei's unbiased gene diversity index, the probability that two randomly selected alleles are different.

*r̄*_d_: the standardized index of association; with a value of zero for a null hypothesis, a population is freely recombining.

Indicates host (P for RGT Planet or S for Spartacus CL), with agricultural zone number.

Indicates the cumulative number of MLGs irrespective of field or region.

**Fig. 2.**
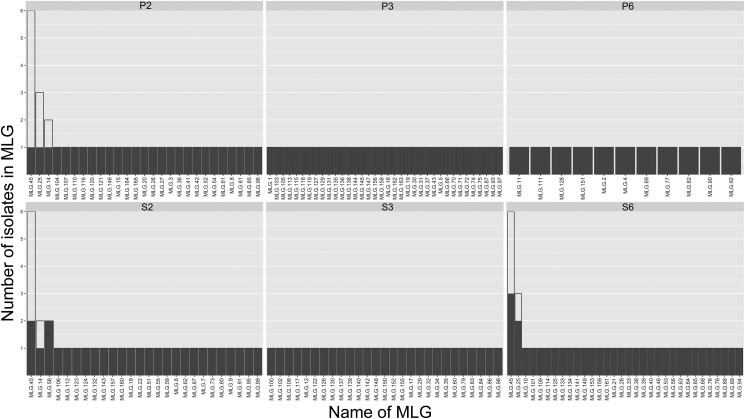
Distribution and size of *Ptm* MLGs across six fields within Western Australia. Number of individuals per MLG per field (solid columns) is shown compared to the total observed individuals per MLG over the six-field sample set (outlined columns).

A rarefaction curve showed no significant deviation in the number of MLGs found within each field based on non-clone-corrected data (Supplementary Fig. 1), while a lack of curve saturation suggested a large number of MLGs exist at each sample site. Four MLGs contained between two and six individuals, with the remaining 151 MLGs consisting of a single representative. MLG 49 was the largest and most geographically widespread group, detected in three fields and comprising six individuals (Fig. 2).

**Fig. 3.**
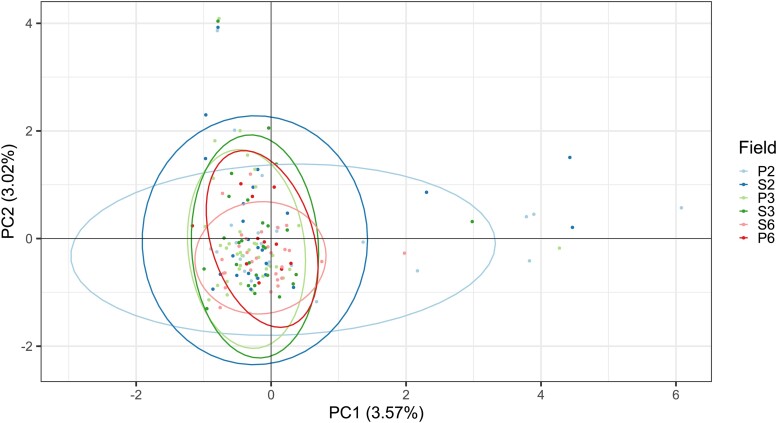
PCA of *Ptm* genotypes from WA field sampling showing the first two principal components. Isolates were collected from three different Agzones and two different host cultivars. The key for individual fields indicate the host cultivar (P for Planet or S for Spartacus) followed by the Agzone number. Ellipses show respective 95% confidence intervals.

Principal components analysis showed no observable population structure when the first two principal components were plotted against one another (Fig. 3). However, unsupervised clustering analysis performed without a priori knowledge of sample location suggested two potential populations (*K* = 2) when 150 principal components were retained. A membership probability plot of each of the fields illustrating the predicted populations suggested weak geographic ties to the predicted clusters (Supplementary Fig. 2), while all six fields contained isolates from cluster one and four contained isolates from cluster two.

**Fig. 4.**
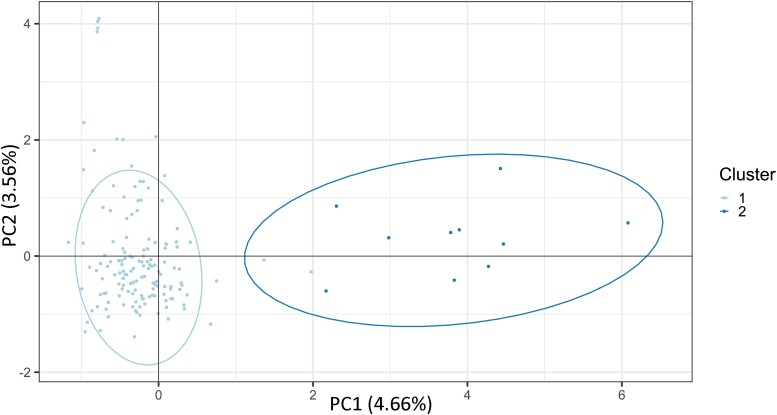
Principal component analysis of putative *Ptm* populations identified by DAPC of isolates collected from individual WA fields. The first two principle components showing differentiation of isolates are shown with 95% population assignment confidence intervals indicated by ellipses.

Further analysis of these two putative populations by AMOVA revealed variation within clusters to be high (∼78%), however, variation between clusters was also moderately high (∼22%). A principle components analysis was performed on the putative two populations and showed significant differentiation (Fig. 4). The corrected Simpson’s index also suggested high genetic diversity within each cluster (1 − *λd* > 0.98).

The role of Agzone origin or isolate host variety in genetic structure was also examined. Similar to the analysis at field level, variances within Agzones and within the same host cv were high and comprised the majority of the total variation (99%), whereas variation between Agzone and host cv was low (1%). MLGs containing multiple individuals were also shared between each Agzone and cv.

### Inter-regional population structure analysis

Isolates collected for inter-regional comparisons were placed into four groups represented by between 8 and 30 individuals based on their origin of collection: Vic, SA, WA, with Qld and NSW combined. The isolates from NSW (*n* = 3) and Qld (*n* = 5) were combined as they represent a contiguous barley-growing area straddling the state border. After clone correction, SNP variance within regions was high and accounted for most of the variation (∼99.1%), whereas variation between the groups was low (∼0.9%). Regional differentiation of isolates was low, similar to that found at the field level (*ϕ* = ∼0.01). Isolates were highly heterogeneous at the regional level, presenting 85 MLGs based on 1278 loci with each MLG confined to a single region (Table 1). Nei's unbiased gene diversity was high for all regions (0.19–0.211), and the corrected Simpson's index suggested high genotype diversity (1 − *λd* > 0.99). Similar to comparisons between groups of isolates collected within fields, each of the four regional groups appeared to be primarily asexually reproducing as portrayed by the *r̄*_d_ distribution.

**Fig. 5.**
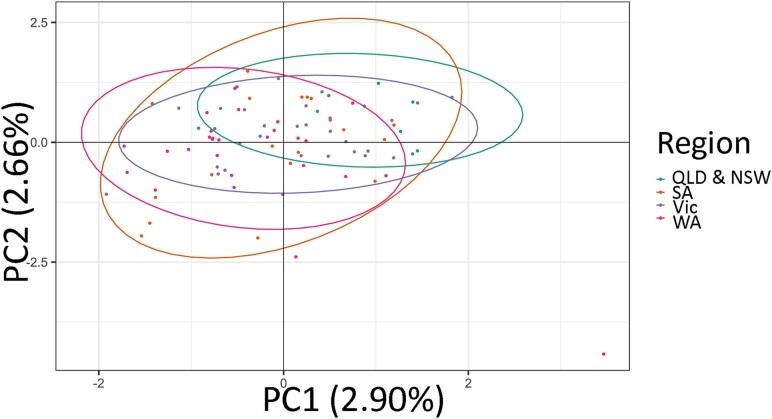
Principal component analysis of all *Ptm* samples from four Australian regions. The first two principal components are shown showing no differentiation of isolates, with ellipses indicating 95% confidence intervals.

PCA eigenvalues calculated for the first two PCAs linking samples to their sample locations accounted for a low ∼6% of the explained variation, with no visible population structure when plotted (Fig. 5). Unsupervised clustering analysis performed without a priori data of sample location suggested the existence of a unique population in Australia (*K* = 1). The Mantel test revealed a small, but positive linear correlation between genetic distance and isolate sample location (*P* = 0.024 and *r* = 0.073), suggesting that despite no detectable genetic structure being present between Australian regions, there is likely a component of isolation by distance (Supplementary Fig. 3).

To increase the data resolution supporting the putative populations established with DAPC in the field level analysis, isolates from both the field level and regional analysis were combined. For this, 239 clone-corrected *Ptm* isolates and 1,271 filtered SNP markers were used, in order to show the total MLG genetic diversity of the four sample regions (Table 2). Genotype diversity was high within each of four national *Ptm* groups and similar to the previous analyses, most of the variation (∼98.3%) occurred within regions, whereas variation between regions was low (∼1.6%), with variations between fields contributing least to genetic variation (∼0.1%).

**Table 2.** Genetic diversity indices of 239 *Ptm* multi-locus genotypes collected from across Australia and their putative DAPC genotypic groups.

	MLG	*H*	1 − *λ*	*H*_exp_	*r̄*_d_
Region					
ȃVic	30	3.40	1.00	0.21	1.0 × 10^−3^
ȃSA	20	3.00	1.00	0.20	2.0 × 10^−3^
ȃQld and NSW	8	2.08	1.00	0.19	2.0 × 10^−3^
ȃWA	181	5.20	1.00	0.21	6.0 × 10^−3^
ȃTotal	239	5.48	1.00	0.21	5.0 × 10^−3^
Cluster					
ȃpop1	227	5.42	1.00	0.20	2.5 × 10^−3^
ȃpop2	12	2.48	1.00	0.19	2.8 × 10^−3^
ȃTotal	239	5.48	1.00	0.21	4.9 × 10^−3^

Data is presented for combined regional *Ptm* collections and of two putative genotypic clusters defined by DAPC, produced within the *poppr* package (Kamvar *et al*. 2014).

MLG: the total number of unique multi-locus genotypes (MLGs) identified per region or genotypic group.

*H*: Shannon–Wiener index of MLG group genotypic diversity, a measure of the number of unique genotypes and their homogeneity.

1 − *λ*: corrected Simpson's index of MLG diversity, the probability that two isolates from the same dataset are different genotypes.

*H*_exp_: Nei's unbiased gene diversity index, the probability that two randomly selected alleles are different.

*r̄*_d_: the standardized index of association; with a value of zero for a null hypothesis, a population is freely recombining.

Unsupervised clustering analysis by PCA performed without a priori knowledge of sample location suggested two potential populations (*K* = 2) (Supplementary Fig. 4). DAPC was used to study the two putative populations with 32 PCA eigenvalues and three discriminant analysis eigenvalues being retained (Supplementary Fig. 5). Notably, one of the populations (pop1) was present in all four regions; however, WA was the only region to contain the second population (pop2). Only one Western Australian isolate from the original regional analysis (19PTX147) contributed to the pop2, the other 11 were isolates from the field level analyses. Genetic diversity indices were high between each putative population in Table 2. Further analysis of the pop1 and pop2 groupings by AMOVA revealed variation within each to be high (∼79%), and the variation between moderately high (∼21%).

Validation of the two cryptic sub-groups with STRUCTURE version 2.3.4 (Pritchard *et al*. 2000) generally supported the DAPC findings, with both analyses showing no geographic ties of the isolates to their regions (Supplementary Fig. 6). However, STRUCTURE indicated the presence of three potential populations rather than the two proposed by DAPC (Supplementary Fig. 7). This third subpopulation of just eight isolates (population assignment >50%) consisted of much more highly related individuals of the larger population identified in DAPC, which was also present only in WA (Supplementary Fig. 8).

### Mating type equilibria

To determine the level of sexual reproduction of *Ptm* in Australia, mating type analyses were performed. PCR amplification of *MAT* loci of the 87 interstate *Ptm* isolates revealed 42 that were *MAT*1–1 and 45 that were *MAT*1–2. Both mating types were present in each of the four state regions did not significantly deviate from a 1:1 ratio for a randomly crossing population in both the original and clone-corrected data (chi-square tests suggested no significant deviation from an expected 1:1 ratio at *P* > 0.05). Similarly, the isolates collected from individual WA fields did not significantly deviate from the 1:1 ratio, both within and between fields, in both the unaltered and clone-corrected data. The clonal isolates in the largest group of clones in MLG: 49, identified among WA isolates, congruently displayed the same mating type, *MAT*1–2 and, as may be expected, all clones within other MLGs amplified the same mating type.

### SillicoDArT markers support lack of population structure and high genotypic diversity in Australia

Dominant SillicoDArT markers were examined independently to DArTSeq SNP markers in complimentary analyses of all regional and field *Ptm* isolates (Supplementary File 1). Results generally agreed with those based on the SNP markers. The SillicoDArT markers distinguished the same MLGs and supported the genetic diversity estimates, with most of the genetic variation (∼98%) occurring within regions, whereas variation between regions was low (∼1.4%), while variation between fields contributed least to genetic variation (∼0.6%). The silicoDArT markers also indicated high genotypic diversity but isolates were closely related.

Unsupervised clustering analysis primarily supported the SNP data, which established a group of isolates clustering separately from the other MLGs (Fig. 4: Cluster 2, Table 2: pop2). Notably, the same WA genotypic group was observed. Clustering analysis however differed in suggesting the existence of three other cryptic subpopulations unrelated to region, Agzone field, or host variety.

### Population differentiating SNP markers are located in genomic regions associated with fungicide resistance

Following DAPC of all clone-corrected isolates, 18 unique markers were associated with differentiation of the two SNP-based genetic clusters (Supplementary Table 2). All the markers mapped by BLASTn to *Ptm* isolate SG1 (BioProject: PRJEB18107, BioSample: SAMEA4560037) within Geneious 8.0.5 (Kearse *et al*. 2012) with *E* values ranging from 3.92 × 10^−30^ to 4.04 × 10^−10^ with >95% identical sites shared. Of the 18 markers, 11 mapped to candidate genes and seven mapped to intergenic regions. The most differentiated marker was found at ∼8500 bp from the fungicide resistance-associated gene *Cyp51A* (GenBank accession: CP060577). Two other markers were also found within 16,000 bp and 31,000 bp of this gene. Two markers were found near to a *Ptr* homologue for a major facilitator superfamily (MFS) gene annotated as a benomyl/methotrexate resistance protein (GenBank accession: XM_001931024).

Orthologous genes in *Ptr* to the 11 SG1 genic markers included a calcium channel protein (GenBank accession: XM_001931060), a tRNA A64-2′-O-ribosylphosphate transferase protein (GenBank accession: XM_001931591), a pumilio domain containing protein (GenBank accession: XM_001931022), low homology to a HC-toxin producing non-ribosomal protein synthase (GenBank accession: XM_001940729), together with other unnamed predicted proteins or conserved hypothetical proteins.

## Discussion

This study combines the largest and most geographically diverse Australian collection of *Ptm* isolates to date with a high-resolution genetic marker technology, enabling an improved resolution of the genetic diversity and relatedness in the pathogen population. Overall, the study confirms high levels of genotype diversity and low levels of clonality. Similar results in both forms of *P. teres* have been observed previously in Australia (Bogacki *et al*. 2010; Ellwood *et al*. 2019) and in other countries such as Sweden (Jonsson *et al*. 2000), Italy (Rau *et al*. 2003), Czech Republic (Leisova *et al*. 2005), Finland (Serenius *et al*. 2007), Canada (Akhavan *et al*. 2016), and Iran (Vasighzadeh *et al*. 2021). Modest levels of genetic recombination were observed both within fields and within national regions. This was shown through linkage disequilibrium analysis using the standardized index of association, *r̄*_d_. For a freely recombining population, the expected score is zero and greater than zero if there is an association between alleles. Both categories had scores greater than zero, indicating limited sexual reproduction with asexual propagation the primary mode. However, equal mating type ratios and low numbers of clonal isolates were also observed, suggesting sexual reproduction is frequent enough to maintain mating type balance.

### Lack of geographic population structure

Isolation by distance and physical barriers to migration commonly contribute to genetic differentiation between individuals. However, despite Australia being some 4000 km wide, geographic clustering of genotypes was absent in this study, with low variation between regions. Wind dispersal of spores provides the most likely explanation. However, the Nullarbor Plain is a mostly treeless, arid area, which stretches over 1000 km and separates WA from the rest of the barley-growing regions in Australia. Despite this, clustering analysis indicated a single Australian population (*K* = 1). Over larger distances, assisted dispersal in infected straw and hay may play a role in suppressing population differentiation, as these are commonly transported between states, particularly during droughts (*Ptm* is not regarded as a significant seed borne pathogen). Another contributing reason may be due to the diseases’ relatively recent introduction into Australia, being first identified in Nabawa, WA in 1977 (Khan 1982), and a lack of time enabling divergence from a founder population. This is a less parsimonious explanation, and assumes similar genotypic lineages have established and persisted throughout the country. Nonetheless, despite the lack of obvious geographic population structure, a Mantel test suggested some isolation by distance, although the effect was low.

Analysis of the population differentiation between fields in WA provided similar results to regional comparisons, with low genetic differentiation between individuals from different Agzones, fields, and the host variety, but a high degree within. This may be expected as, unlike comparisons of genotypes between different regions defined by clear geographic boundaries, there are extensive barley-growing regions in WA from Agzone 2 through to Agzone 6. Furthermore, the distribution of MLG groups indicated neither Agzone nor host cv appeared to play a role in genotypic selection among the isolates. The cvs the isolates were sampled from, RGT Planet and Spartacus CL, were independently developed by different breeding companies. Both are rated as susceptible to SFNB, and the *Ptm* genotypic compositions on these hosts do not indicate independent gain of virulence. These have been the most popular varieties sown in WA in recent years, whereby Spartacus CL was the most common variety in 2019 and 2020 with RGT Planet in second position, together accounting for nearly 70% of the area sown (Paynter *et al*. 2022). As other popular cvs are predominantly susceptible, the detection of gain of virulence by *Ptm* in Australia might be regarded as the exception. However, anecdotal reports suggest *Ptm* is capable of defeating SFNB resistance. For example in WA, Thomas *et al*. (2018) noted the erosion of partial seedling resistance in cvs Scope and Hindmarsh, while seedling resistance was overcome in cv Baudin (Muria-Gonzalez *et al*., manuscript submitted).

In the most intensively sampled region, WA, just three MLGs were found with individuals distributed across sites used for field level sampling at locations over 650 km apart. The low number of clones in these MLGs (11) provides weak evidence for recent selection of beneficial genotypes, perhaps masked by susceptible popular cvs. For example, predominantly clonal *Ptm* isolates were previously found in cv Oxford in southern WA, associated with a virulent new pathotype in combination with demethylase inhibitor (DMI) fungicide resistance (Turo *et al*. 2021). The transience of the genetic background of clonal expansions in *Ptm* may be similar to *Ptt*, where DMI resistance was rapidly assimilated into the wider population (Ellwood *et al*. 2019).

### Genotypic clustering is independent of geographic origin

Clustering analysis by DAPC suggested the presence of two populations (*K* = 2). These were not linked to the field of origin, with individuals present across most of the sampled fields, potentially suggesting a recent introduction or selection of new genotypes variants in WA. Bayesian analysis with STRUCTURE suggested the presence of three populations (*K* = 3). The two populations identified by DAPC were supported, with a third composed of more highly related individuals representing a subset of the larger DPAC pop1 group. This population was present only in WA and may represent selection of an advantageous genotype, and contains MLG: 49, the largest group of clones found in WA. However, despite the removal of isolates sharing the same MLG prior to STRUCTURE analysis, the validity of this group should be assessed in the context of the limitations of the STRUCTURE algorithm, which requires populations to be in Hardy–Weinberg equilibrium.

A lack of Australian *Ptm* geographic population structure is in agreement with a study by McLean *et al*. (2014), which assessed the genetic structure of *Ptm* in Australia using a geographically diverse set of isolates collected between 1996 and 2009 (*n* = 60). The authors also identified two genetically distinct *Ptm* clusters unlinked to sampling year and sampling region, however, in that study an Australia-wide population structure was not established. Our study confirms the presence of a single Australia-wide *Ptm* population, with one or more smaller populations unrelated to sampling site, referred to here as cryptic populations.

Our results also complement aspects of other *Ptm* genetic diversity studies conducted within Australia. Bogacki *et al*. (2010) found that isolates sampled from different areas of the same field and between fields were genetically similar and Serenius *et al*. (2007) found that the majority of the genetic differentiation of *Ptm* occurred within fields rather than between fields or regions. However, these two studies differ in their degree of differentiation, Bogacki *et al* (2010) finding a very low degree of differentiation between *Ptm* field populations in line with the results of this study whereas Serenius *et al*. (2007) finding a much larger degree between states and fields (21.94 and 11.24%, respectively) with low variation within fields (66.82%). In this study, 98% if the genetic variation occurred within fields and only 1.6% occurred between fields and/or regions.

There are different explanations for the contrasting results between our study and previous research; greater distances between sampling sites should mean less migration, and therefore larger differences should be found between sampling locations than within the same fields. However, when comparing fields from Agzone 2 and Agzone 6, which are 650 km apart from each other, genetic diversity within fields is significantly higher than between fields. A more likely explanation for the differing results is the molecular marker technologies used to differentiate isolates, with the major factor affecting resolution appearing to be a comparatively low number of genetic markers in the earlier studies.

### Genotypic clustering is associated with fungicide resistance

Three of the markers identified by DAPC associated with population differentiation were located on Chromosome 6, within 31 Kbp of the DMI fungicide resistance-associated gene *Cyp51A* (Mair *et al*. 2020) and two of these markers contributed most to differentiation. A second group of markers implicated with fungicide resistance were located within 3 Kb of a *Ptr* orthologue encoding a benomyl/methotrexate resistance-like protein, and may indicate an alternate mode of fungicide resistance in *Ptm*. Related genes are implicated in multi-drug resistance (MDR) and are part of the MFS. MDR transporters are proton antiporters that mediate the efflux of a diverse range of drugs and toxic compounds. For example, they have been implicated in providing resistance to quinidine (Vargas *et al*. 2007), amiloride (Stolz *et al*. 2005), and fluconazole (Keniya *et al*. 2015) in yeast. In the wheat pathogen, *Zymoseptoria tritici*, MDRs provide enhanced fungicide resistance to tolnaftate, terbinafine, the DMI metconazole, the quinone outside inhibitor azoxystrobin, and the succinate dehydrogenase inhibitor boscalid (Omrane *et al*. 2017). These marker associations concur with an international study of *Ptt*, where markers nearby to *Ptt Cyp51A* and a MFS domain-containing protein were also implicated in underlying population structure (Dahanayaka *et al*. 2021).

Other markers that were associated with genetic structuring included a predicted *Ptm* non-ribosomal peptide synthase (NRPS) gene that showed homology to a *Ptr* gene annotated as HC-toxin. HC-toxins are a form of host-selective toxins that are thought to operate through the prevention of defense gene expression. For example, HC-toxin is a tetrapepetide produced by *Cochliobolus carbonum*, which inhibits histone deacetylase leading to disease symptoms on susceptible plants (Brosch *et al*. 1995). However, the predicted product of the *Ptm* gene in question is a likely a pentapeptide based on the number of NRPS gene modules, therefore the function may be different. Other cyclic pentapeptide host-selective toxins such as victorin, produced by *Cochliobolus victoriae*, are known to exist, however these are not currently considered NRPSs (Kessler *et al*. 2020). NRPSs also produce other bio-active secondary metabolites, such as those involved in cellular development and stress response (Keller *et al*. 2005). The possible role of this gene in differential virulence of *Ptm* isolates may warrant further examination in future studies.

Additional *Ptm* genes orthologous to annotated *Ptr* genes included: A calcium channel protein 1 (*CCH1*), which may have a role in homeostasis and virulence in *Aspergillus fumigatus (*de Castro *et al*. 2014*)*; tRNA A64-2′-O-ribosylphosphate transferase protein, which modifies cytoplasmic initiator tRNAs, preventing them from participating in translational elongation (Kiesewetter *et al*. 1990); finally, a pumilio domain-containing protein, implicated in post-transcriptional regulation influencing mRNA stability, translation, and localization (Wang *et al*. 2018).

This study has revealed a single Australia-wide population of *Ptm* suggesting unconstrained movement of genotypes within Australia. There also appears to be a lack of recent directional selection although we detected cryptic populations. This is consistent with the most popular barley varieties being susceptible to SFNB (Paynter *et al*. 2022), while the cryptic populations appear to be related to generalized adaptions, in particular the widespread application of fungicides, but may include other environmental or biotic stresses.

Incorporating new resistance genes into modern cvs is the most cost-effective approach to combatting serious crop diseases such as SFNB. This research is of significance to breeders and growers alike, as long-term control measures require an improved understanding of plant–pathogen interactions. *Ptm* is genotypically diverse and mobile pathogen, underlying a need for nationally coordinated disease management and resistance breeding strategies. The challenge to breeders in developing new varieties will be in extensive testing against the range of known pathotypes and in deploying resistance genes combinations that reduce the likelihood of their breakdown.

## Supplementary Material

jkad076_Supplementary_Data

## Data Availability

Supplemental Material included at figshare: https://doi.org/10.25387/g3.21611043. Supplementary Table 1 contains DArTseq SNP and dominant markers together with metadata descriptions and is hosted at: https://figshare.com/s/c2dcabcf2351a9043e30. Supplementary Table 2 gives isolate collection site data, forma and mating type of *P. teres* isolates. Supplementary Table 3 gives *Ptm* DAPC SNP markers contributing to population structure and annotations of corresponding orthologous genes in *Ptr*. SilicoDArT analyses are provided in File S1. Supplementary Figures 1–8 are combined in a single file with the following contents: Supplementary Fig. 1. Rarefaction curve analysis describing observed *Ptm* MLG richness. Supplementary Fig. 2. Membership probability plot showing the population assignment for each of the 155 field isolate MLGs and their respective collection sites. Supplementary Fig. 3. Isolation by distance plot illustrating the pattern of genetic differentiation among *Ptm* genotypes collected from four Australian regions. Supplementary Fig. 4. Discriminant analysis of principal components (DAPC) cluster analysis of Australian *Ptm* isolates. Supplementary Fig. 5. Scatter plot of the first two PCA principle components resolving Australian *Ptm* isolates. Supplementary Fig. 6. Bayesian cluster analysis of Australian *Ptm* genotypes. Supplementary Fig. 7. Structure Harvester cluster analysis of Australian *Ptm* isolates. Supplementary Fig. 8. Scatter plot of the first two principle components based on data from STRUCTURE 2.3.4.
