# Supplementary material for: Widespread genetic heterogeneity and genotypic grouping associated with fungicide resistance among barley spot form net blotch isolates in Australia
Source: G3 (Bethesda). 2023 Apr 1;13(5):jkad076. doi: 10.1093/g3journal/jkad076 (PMC10151411; doi:10.1093/g3journal/jkad076)
Supplement: jkad076_Supplementary_Data [file jkad076_supplementary_data.zip › File S1.docx]

# File S1. SilicoDArT analyses

Data analysis and quality control method was similar to that performed with the DArTSeq SNP data, with markers retained with a minimum reproducibility score of one and removing markers with >5% missing data. Following filtering, 1097 markers remained from 6321.

The same isolates used in the DArTseq SNP analysis were used in the sillicoDArT study. Two hundred and fifty-one isolates were classified into 239 MLGs as with the DArTseq SNP markers. All MLGs were found unique to a single state and only WA was found to consist of isolates sharing an MLG (Supplementary analysis table 1.1). The sillicoDArT analysis also supported the DArTseq SNP genetic diversity tests and revealed most of the genetic variation (~98%) occurred within regions whereas variation between regions was low (~1.4%), with variations between fields contributing least to genetic variation (~0.6%). The silicoDArT markers also suggested high genotypic diversity but overall isolates were closely related, supporting the DArTSeq SNP marker data.

**Supplementary analysis Table 1.** **Genetic diversity of 251 *Ptm* isolates collected from across Australia based on sillicoDArT markers.** Summary statistics were produced with the poppr command in poppr package in RStudio (RStudio, Boston, Massachusetts, USA).

| **Region** | ***n*** | **MLG** | ***H*** | **1-λ** | ***H_exp_*** | **r̄_d_** |
| --- | --- | --- | --- | --- | --- | --- |
| Vic | 30 | 30 | 3.4 | 1 | 0.188 | 0.012 |
| SA | 20 | 20 | 3 | 1 | 0.175 | 0.013 |
| Qld and NSW | 8 | 8 | 2.08 | 1 | 0.193 | 0.005 |
| WA | 193 | 181 | 5.15 | 0.993 | 0.189 | 0.015 |
| Total | 251 | 239 | 5.44 | 1 | 0.190 | 0.013 |

*n*: Number of isolates in a sample group after data quality filtering.

MLG: The total number of unique multi-locus genotypes (MLGs) identified per region. *Indicates the cumulative number of MLGs irrespective of region.

*H*: Shannon-Wiener index of MLG group genotypic diversity, a measure of the number of unique genotypes and their homogeneity.

1- λ: Corrected Simpson’s index of MLG diversity, the probability two isolates from the same dataset are different genotypes.

*H_exp_*: Nei’s unbiased gene diversity index, the probability that two randomly selected alleles are different.

r̄_d_: The standardised index of association, with a value of zero for a null hypothesis a population is freely recombining.

Principal components analysis showed no observable population structure related to collection regions when the first two principal components were plotted against one another (Figure 1). However, unsupervised clustering analysis performed without *a priori* knowledge of sample location suggested four potential populations (K = 4, Figure 2). DAPC was then used to study the four putative populations with 40 PCA eigenvalues and two discriminant analysis (DA) eigenvalues being retained. Notably, three of the populations (pop1, pop3 and pop4) were present in all four regions; however, Western Australia was the only region to contain the second population (pop2). Only one Western Australian isolate from the original regional analysis (19PTX147) contributed to the pop2, the other ten were isolates from the field level analyses. Isolates in pop2 were also found in pop2 two in the field and regional combined analysis with the SNP markers.


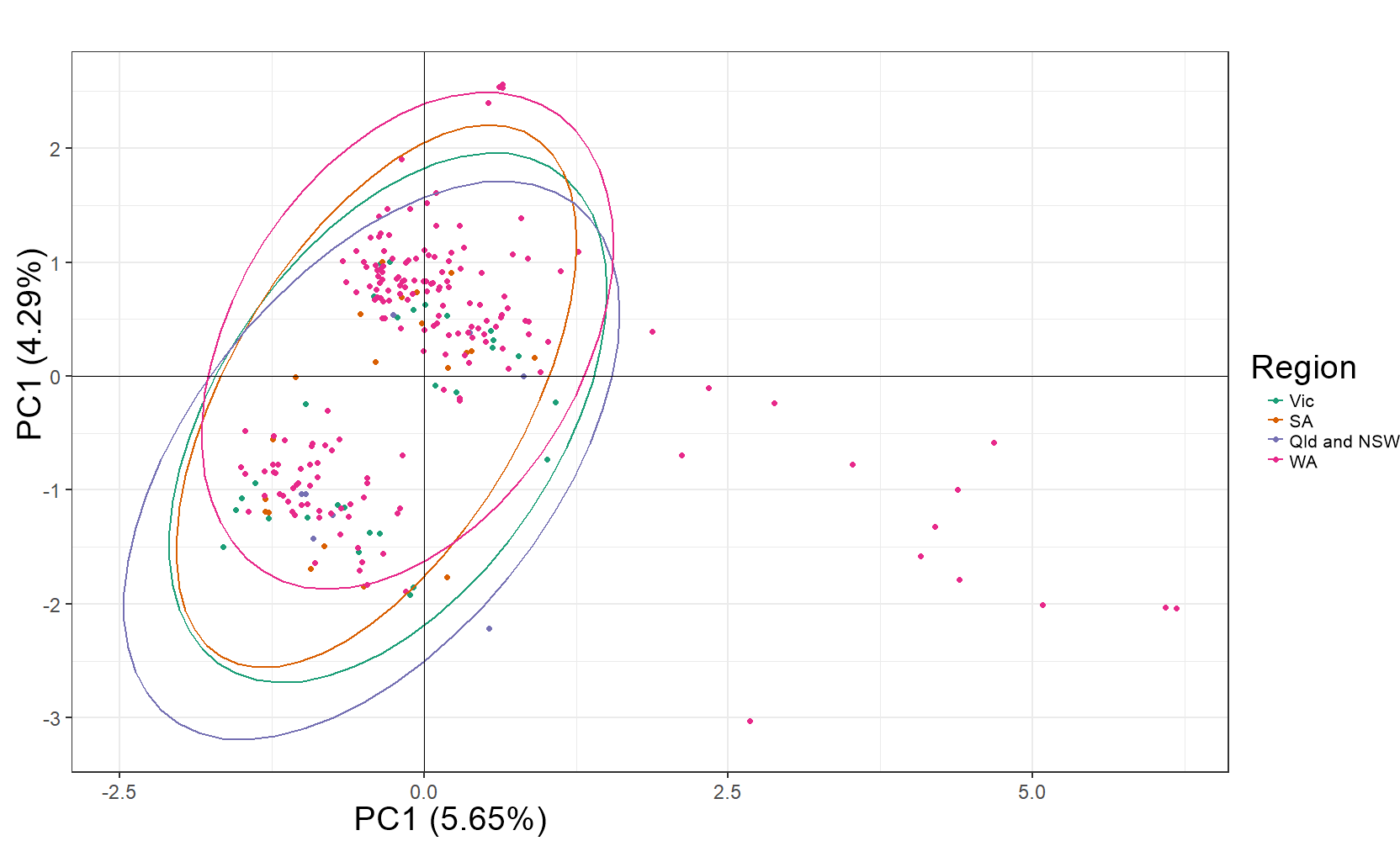


**Supplementary analysis Fig 1. Principal component analysis of all *Ptm* samples from four Australian regions.** The national groups are represented by purple, orange, green and pink dots, Respective coloured eclipses indicate 95% confidence intervals.


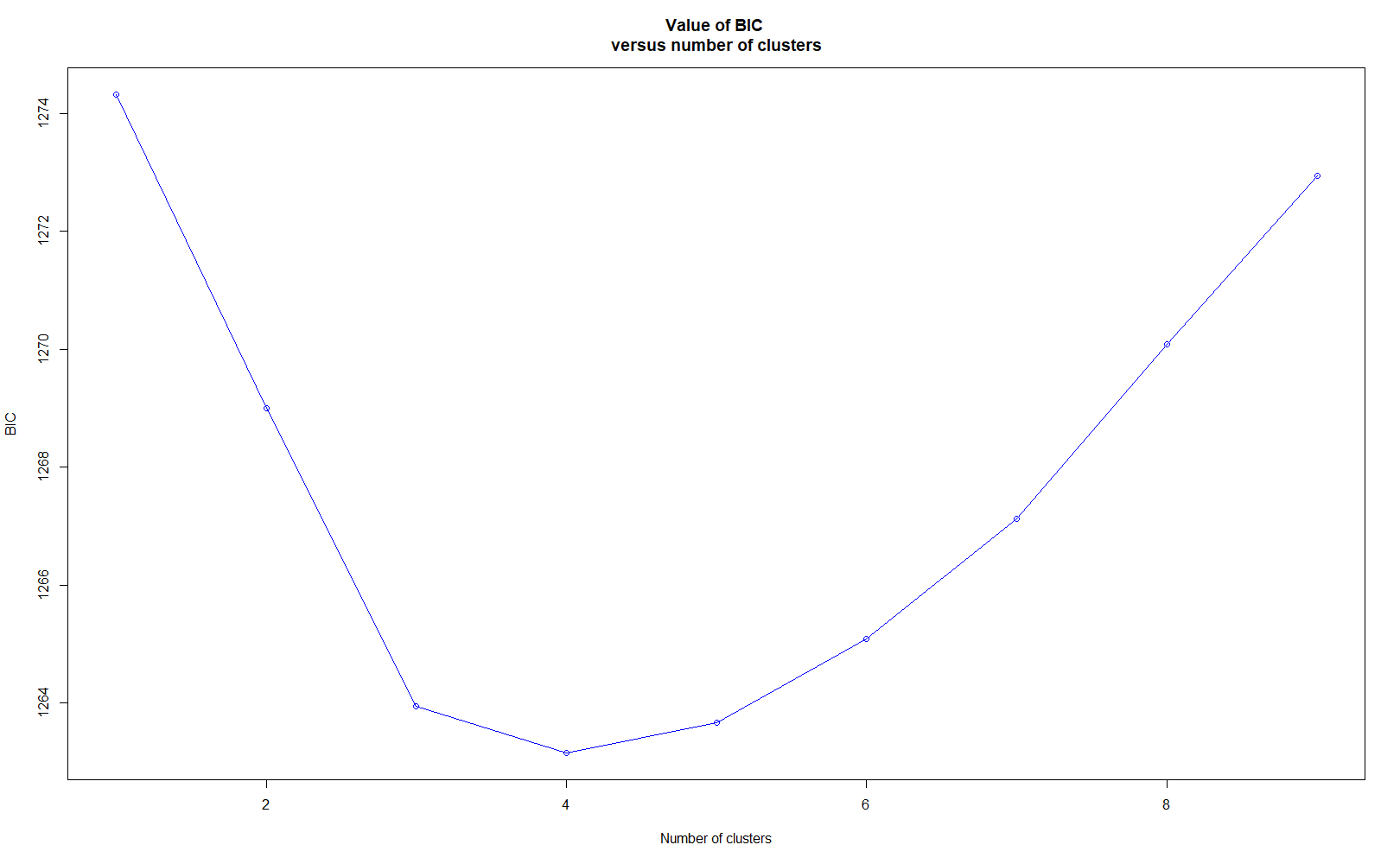


**Supplementary analysis Fig 2. Cluster analysis using sillicoDArT markers based on DPAC (DAPC, Jombart et al. 2010) with 251 Australian *Ptm* isolates collected.** In order to produce the line plot, successive runs of K-means were recorded with an increasing number of clusters (K), after transforming the isolate sillicoDArT data using a principal component analysis (PCA). For each cluster, a BIC (Bayesian information criterion) value is computed. Using the ‘elbow method’ the optimal K value was inferred as four. The procedure was performed in RStudio (RStudio, Boston, Massachusetts, USA) using the ‘kmean’ function as part of the ‘*stats*’ package.
