# Supplementary material for: Widespread genetic heterogeneity and genotypic grouping associated with fungicide resistance among barley spot form net blotch isolates in Australia
Source: G3 (Bethesda). 2023 Apr 1;13(5):jkad076. doi: 10.1093/g3journal/jkad076 (PMC10151411; doi:10.1093/g3journal/jkad076)
Supplement: jkad076_Supplementary_Data [file jkad076_supplementary_data.zip › Supplemental Figures.docx]

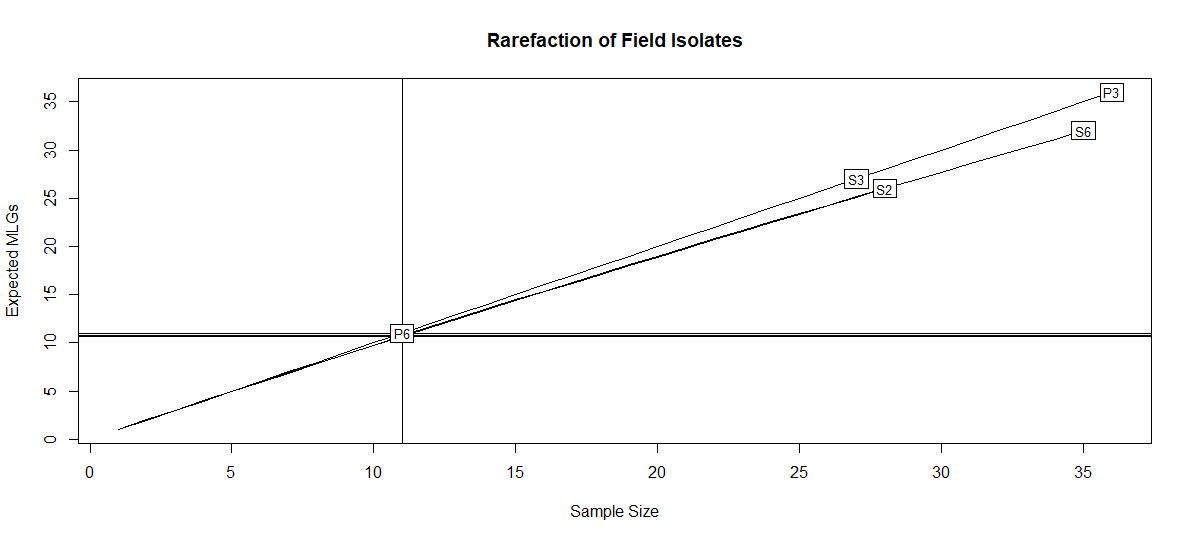


**Figure S1.** **Rarefaction curve analysis describing observed *Ptm* MLG richness.** The plot shows the number of observed multi-locus genotypes from the six WA *Ptm* field collections compared to the expected number. Sites P2 and S3 have an identical number of MLGs and samples (Table 1) and are labelled at the same position. The straight lines indicate that sampling did not achieve saturation of the potential number of MLGs. Plot rendered in RStudio (RStudio, Boston, Massachusetts, USA) using the ‘*rarecurve*’ command in R package ‘*vegan*’ package.


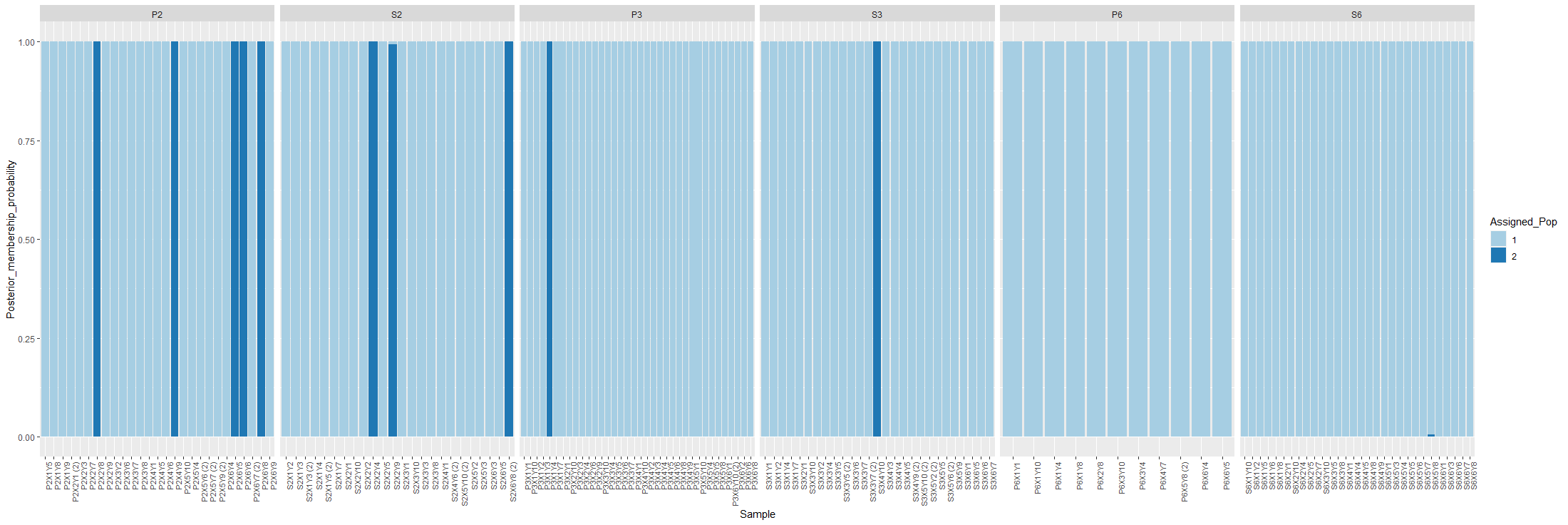


**Figure S2.** **Membership probability plot showing the population assignment for each of the 155 field isolate MLGs and their respective collection sites.** Each genotype is represented by a single vertical bar, typically broken into coloured segments, with lengths proportional to membership of K inferred genetic clusters based on DAPC (although the samples above are predominantly assigned to a single cluster). The plot was produced in RStudio (RStudio, Boston, Massachusetts, USA) using DAPC population assignment data and the ‘*compoplot*’ command as part of the ‘*adegenet’* package.


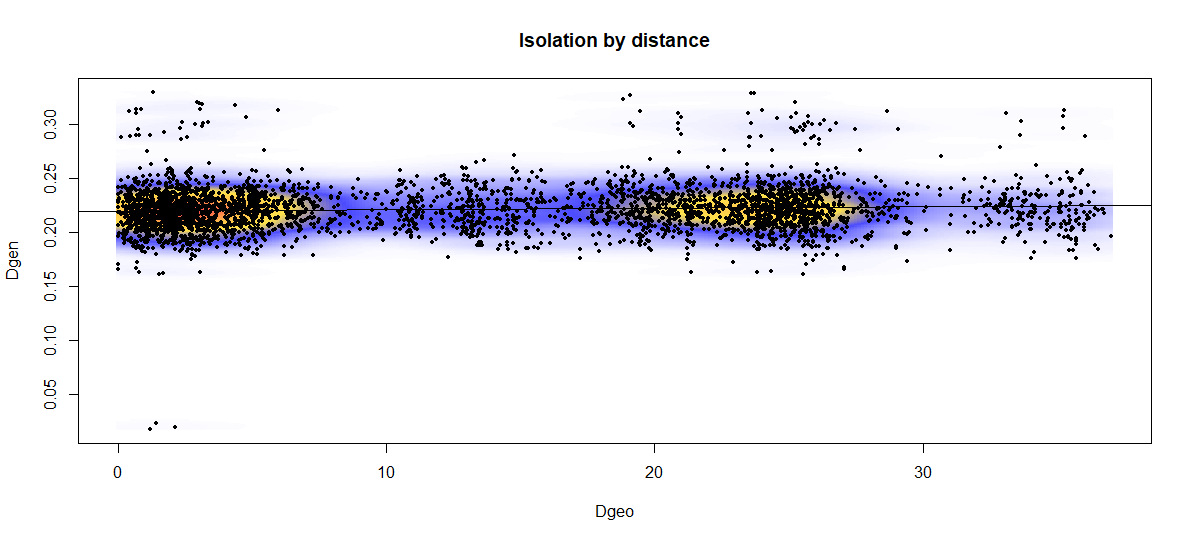


**Figure S3. Isolation by distance plot illustrating the pattern of genetic differentiation among *Ptm* genotypes collected from four Australian regions.** Geographic distance is plotted on the x-axis and genetic distance is plotted on the y-axis. A low but significant amount of correlation of genetic distance with geographic distance is described by the black line (*p* = 0.024 and r = 0.073). The plot was produced in RStudio (RStudio, Boston, Massachusetts, USA) using the ‘*mantel.rtest’* command in the ‘*ade4’* package.


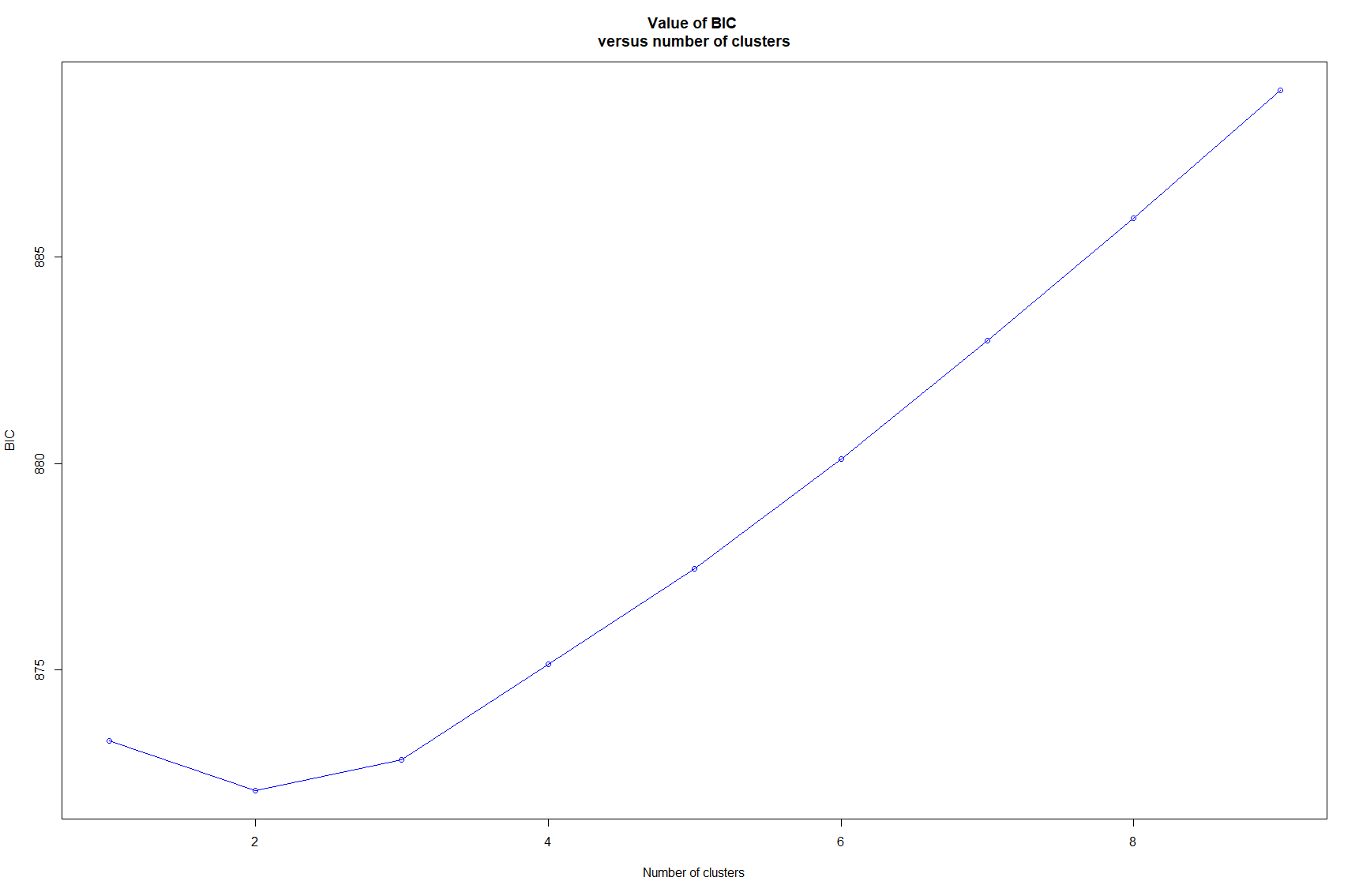


**Figure S4. Discriminant analysis of principal components (DAPC) cluster analysis of Australian *Ptm* isolates.** DAPC (Jombart *et al*. 2010) was performed using 239 *Ptm* genotypes and 1271 SNP markers from both the field and regional collections. In order to produce the line plot, successive runs of K-means are recorded with an increasing number of clusters (K), after transforming the isolate SNP data using a principal component analysis (PCA). For each cluster, a BIC (Bayesian information criterion) value is computed and the optimal K value inferred from the ‘elbow’ inflection, here depicted as two. The procedure was performed in RStudio (RStudio, Boston, Massachusetts, USA) using the ‘kmean’ function as part of the ‘*stats*’ package.


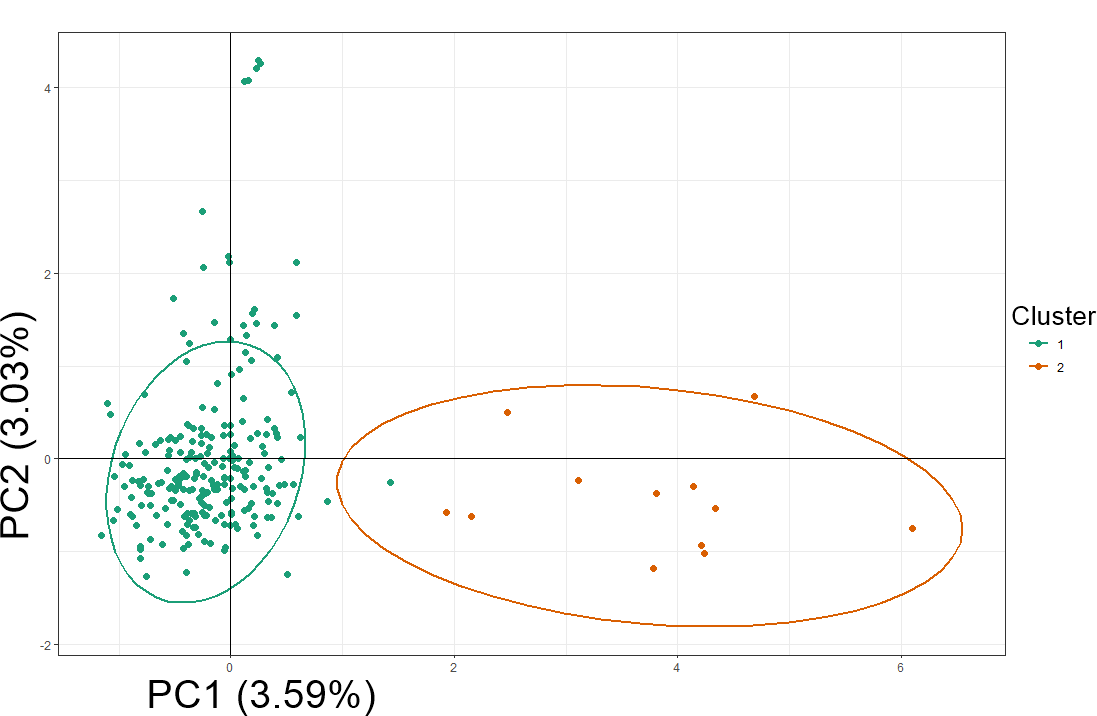


**Figure S5.** **Scatter plot of the first two PCA principle components resolving Australian *Ptm* isolates.** The plot shows the two genotypic clusters identified by DAPC in Supplementary Figure 4. The clusters are represented by green and orange dots with 95% confidence intervals indicated by respective coloured ellipses. Figure produced in RStudio (RStudio, Boston, Massachusetts, USA) with PCA scores generated using the ‘*glPCA*’ command in the adegenet package.


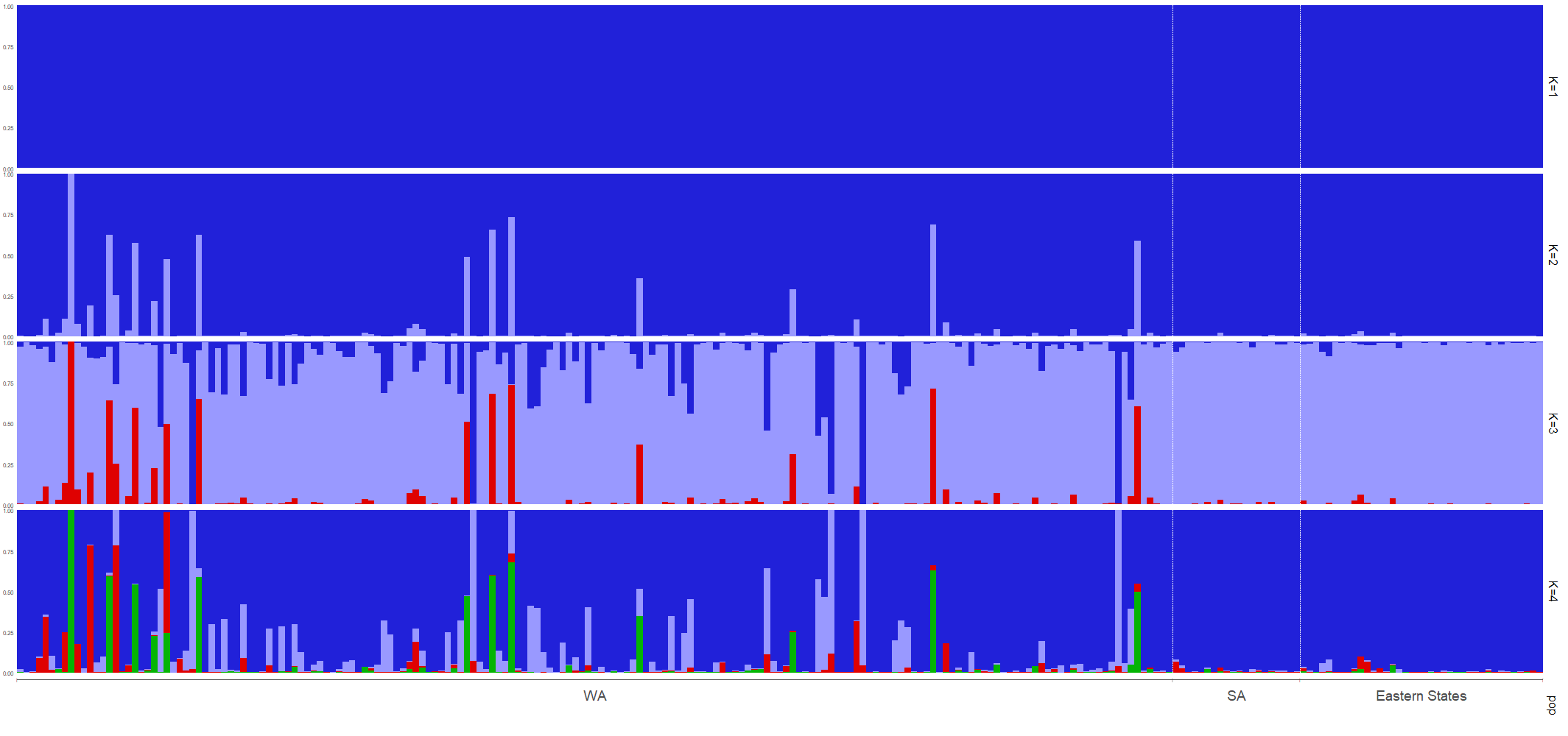


**Figure S6. Bayesian cluster analysis of Australian *Ptm* genotypes.** STRUCTURE 2.3.4 (Pritchard *et al*. 2000) output depicting genotypic grouping inference based on 239 Ptm genotypes and 1271 SNP markers from both the field and regional collections. K = 1-4 is depicted, with each genotype represented by a single vertical bar broken into coloured segments, with lengths proportional to membership of K inferred genetic clusters. K = 2 was supported by DAPC in Supplementary Figure 4. K = 3 was supported by the Evanno ∆k method in Supplementary Figure 7. Isolates are grouped by geographic region on the x-axis and ‘Eastern States’ isolates refers to isolates from Victoria, Queensland and NSW.


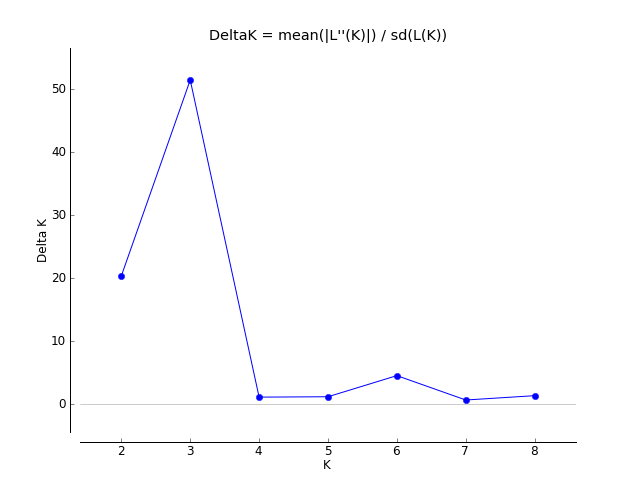


**Figure S7. Structure Harvester cluster analysis of Australian *Ptm* isolates.** The online software Structure Harvester (Earl and von Holdt 2012) was used to determine the most likely number of subpopulations based on Q-value outputs from STRUCTURE v 2.3.4 using the Evanno ∆k method (Evanno, *et al*., 2005). The most optimal number of clusters inferred in this model is three and is based on 239 Ptm genotypes and 1271 SNP markers from both the field and regional collections. The Evanno ∆k method uses the greatest change in magnitude of the second-order rate of change in ln Pr(X|K) against successive K values to indicate the most optimal K value (Gilbert, 2016).


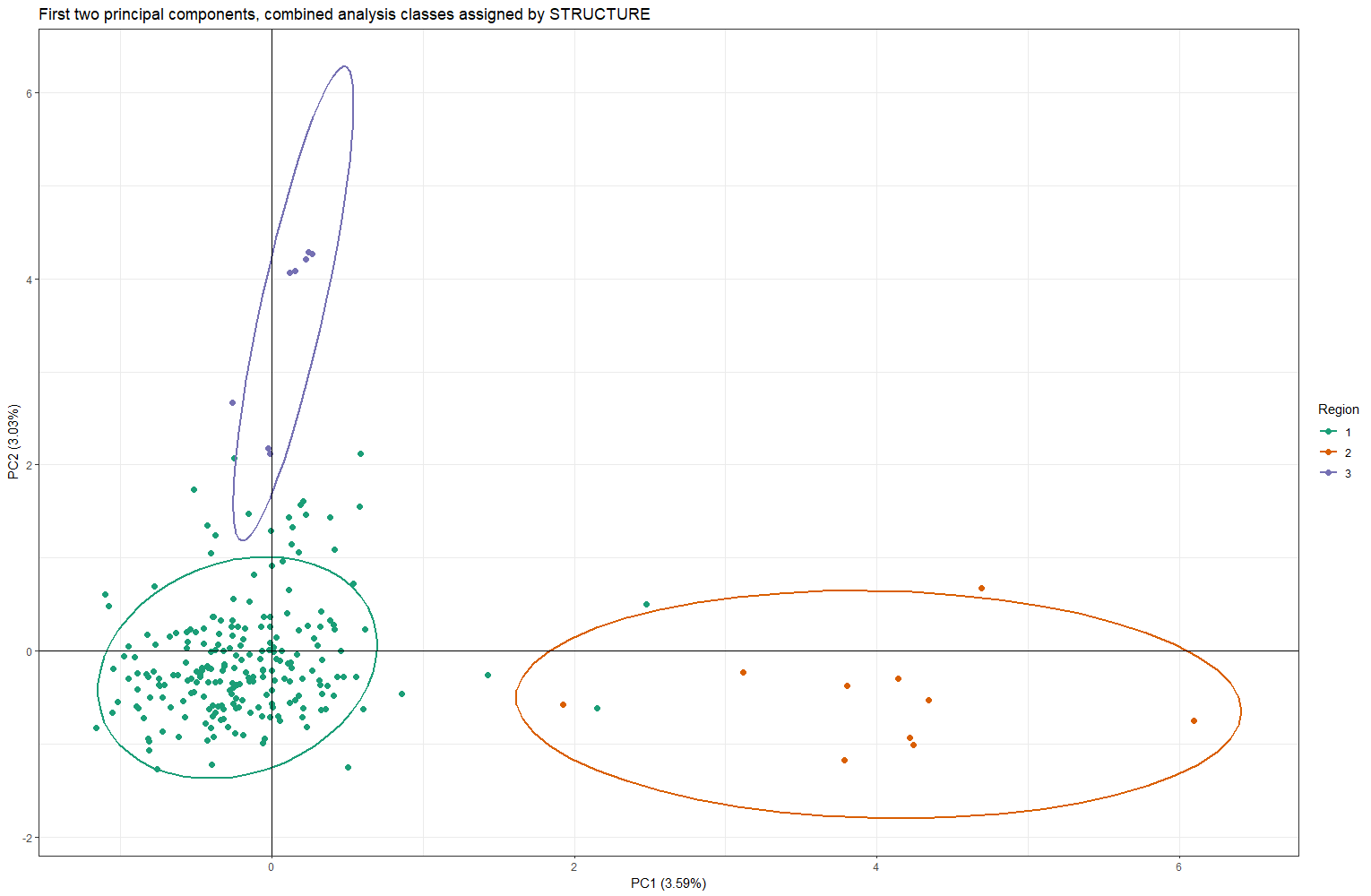


**Figure S8.** **Scatter plot of the first two principle components based on data from STRUCTURE 2.3.4.** Structure Harvester (Earl and von Holdt 2012) indicated K=3 as the most likely number of genetic clusters (Supplementary Figure 7). The clusters are represented by green, orange and purple dots, with 95% confidence intervals indicated by respective coloured ellipses. Image rendered in RStudio (RStudio, Boston, Massachusetts, USA) with PCA scores produced using the ‘*glPCA*’ command in the adegenet package.

# References:

Earl, D. A., & vonHoldt, B. M. (2011). STRUCTURE HARVESTER: a website and program for visualizing STRUCTURE output and implementing the Evanno method. *Conservation genetics resources, 4*(2), 359-361. doi:10.1007/s12686-011-9548-7

Evanno, G., Regnaut, S., & Goudet, J. (2005). Detecting the number of clusters of individuals using the software STRUCTURE: a simulation study. *Mol Ecol, 14*(8), 2611-2620. doi:10.1111/j.1365-294X.2005.02553.x

Gilbert, K. J. (2016). Identifying the number of population clusters with structure: problems and solutions. *Molecular Ecology Resources, 16*(3), 601-603. doi:https://doi.org/10.1111/1755-0998.12521

Jombart, T., Devillard, S., & Balloux, F. (2010). Discriminant analysis of principal components: a new method for the analysis of genetically structured populations. *BMC Genetics, 11*(1), 94. doi:10.1186/1471-2156-11-94

Pritchard, J. K., Stephens, M., & Donnelly, P. (2000). Inference of population structure using multilocus genotype data. *Genetics, 155*(2), 945-959. doi:10.1093/genetics/155.2.945

RStudio Team (2020). RStudio: Integrated Development for R. RStudio, P., Boston, MA URL http://www.rstudio.com/.
